# Converted laparoscopic distal pancreatectomy: is there an impact on patient outcome and total cost?

**DOI:** 10.1007/s00423-021-02427-y

**Published:** 2022-02-07

**Authors:** Riccardo Casadei, Carlo Ingaldi, Claudio Ricci, Emilio De Raffele, Laura Alberici, Francesco Minni

**Affiliations:** 1 Division of Pancreatic Surgery, IRCCS, Azienda Ospedaliero Universitaria di Bologna, Bologna, Italy; 2 Department of Internal Medicine and Surgery (DIMEC), Alma Mater Studiorum, University of Bologna, S.Orsola-Malpighi Hospital, Via Massarenti n.9, 40138 Bologna, Italy

**Keywords:** Laparoscopic, Open, Distal pancreatectomy, Propensity score matching

## Abstract

**Purpose:**

Recent studies have reported worse outcomes of converted laparoscopic distal pancreatectomy (CLDP) with respect to total laparoscopic (TLDP) and open (ODP). The aim of the study was to evaluate the impact of conversion on patient outcome and on total cost.

**Methods:**

Patients requiring a conversion (CLDP) were compared with both TLDP and ODP patients. The relevant patient- and tumour-related variables were collected for each patient. Both intra and postoperative data were extracted. Propensity score matching (PSM) analysis was carried out to equate the groups compared.

**Results:**

Two hundred and five patients underwent DP, 105 (51.2%) ODPs, 81 (39.5%) TLDPs, and 19 (9.3%) CLDPs. After PSM, 19 CLDPs, 38 TLDPs, and 38 ODPs were compared. Patients who underwent CLDP showed a significantly longer operative time (*P* < 0.001), and an increase in blood loss (*P* = 0.032) and total cost (*P* = 0.034) with respect to TLDP, and a significantly longer operative time (*P* < 0.001), less frequent postoperative morbidity (*P* = 0.050), and a higher readmission rate (*P* = 0.035) with respect to ODP.

**Conclusion:**

Total laparoscopic pancreatectomy was superior regarding operative findings and total costs with respect to CLDP; ODP showed a higher postoperative morbidity rate and a lower readmission rate with respect to CLDP. However, the reasons for the readmission of patients who underwent CLDP were mainly related to postoperative pancreatic fistula (POPF) grade B which is usually due to pancreas texture. Thus, the majority of distal pancreatectomies can be started using a minimally invasive approach, performing an early conversion if necessary.

## Introduction

Laparoscopic distal pancreatectomy (LDP) is becoming the standard treatment for patients with left-sided pancreatic tumours from both a clinical and quality-of-life point of view [1]. However, it represents a challenging procedure with different degrees of technical complexity and a high conversion rate ranging from 6.5 to 27.3% [2–6]. The reasons for the high conversion rate have to be explained, and the safety and feasibility of the laparoscopic approach may be questioned in selected cases as conversion may have a negative effect on patient outcome. In fact, recent studies have reported worse results for converted laparoscopic distal pancreatectomy (CLDP) with respect to total laparoscopic distal pancreatectomy (TLDP) and planned open distal pancreatectomy (ODP) [3, 7–10]. The aim of the current study was to evaluate the effects of conversion on patient outcome and on the total cost of the surgical procedures by comparing CLDP versus TLDP and ODP using a propensity score matching (PSM) analysis. The primary endpoint was to evaluate the overall complication rate. The secondary endpoints included the assessment of operative findings (operative time, blood loss), postoperative outcomes (90-day mortality, severe postoperative morbidity, clinically relevant postoperative pancreatic fistula [CR-POPF], POPF grade C, post-pancreatectomy haemorrhage [PPH], delayed gastric emptying [DGE], reoperation rate, readmission rate, length of hospital stay), and the total cost of the surgical procedures.

## Material and methods

### Study design

A single tertiary referral centre retrospective cohort study based on a prospectively maintained database, of patients undergoing distal pancreatectomy (DP) for body-tail pancreatic neoplasms from January 2005 to January 2021 was carried out. The study was approved by the Ethical Committee of S. Orsola-Malpighi Hospital with code PANBO 064/2017/ U/Oss; patient informed consent was obtained from all participants included in the study. The patients who underwent DP were divided into three groups: ODP, TLDP, and CLDP. Patients requiring a conversion (CLDP) were compared with both TLDP and ODP patients. A comparison between TLDP and ODP was not carried out. For each patient, the following relevant variables were collected: (1) patient-related (gender, age, comorbidities, American Society of Anesthesiologists (ASA) score, body mass index (BMI), previous abdominal surgery, and extended procedures) and (2) tumour-related (tumour size, tumour site, and malignancy). The malignant tumours included pancreatic ductal adenocarcinomas (PDACs); the non-malignant tumours included intraductal papillary mucinous neoplasms (IPMNs), mucinous and serous cystadenomas, and pancreatic neuroendocrine tumours (pNETs). The intra- and postoperative data (operative time, blood loss, 90-day mortality, postoperative morbidity, severe postoperative morbidity, CR-POPF, postoperative pancreatic fistula grade C, PPH, DGE, reoperation rate, readmission rate, length of hospital stay, and the total cost of the procedures) were also extracted.

### Terminology and definitions

When the authors indicated that the tumour was in the body of the pancreas, it signified that it was located between the left border of the portal vein and the left border of the aorta; when the authors indicated that the tumour was located in the pancreatic tail, it signified that it was distal to the left border of the aorta. A left distal pancreatectomy was defined as the transection of the pancreas on the left border of the portal vein; a subtotal distal pancreatectomy was defined as the transection of the pancreas on the right border of the portal vein. In a subtotal distal pancreatectomy, the resection line was at the level of the portal vein, requiring a tunnelling procedure, while in a left pancreatectomy, the tunnelling procedure was not required. The pancreatic transection was always performed using a stapler. An extended procedure was defined as a surgical resection involving other neighbouring organs in addition to the pancreas. A converted laparoscopic distal pancreatectomy was defined as any resection which was attempted as laparoscopic, but required conversion thereafter with any laparotomy or hand assistance for reasons other than trocar placement or specimen extraction [11]. A total laparoscopic distal pancreatectomy was considered to be a successfully completed laparoscopic distal pancreatectomy. Operative time was defined as the interval from the incision to the suturing of the skin. Poor visualisation of the tumour meant that the tumour was not clearly identified, even if an intraoperative ultrasound was performed. Postoperative mortality was defined as the number of deaths occurring during hospitalisation or within 90 days after surgery. Postoperative morbidity included all complications following surgery up to the day of discharge according to the Clavien-Dindo classification [12]. Major complications were classified as Clavien-Dindo > 2 [13]. A postoperative pancreatic fistula (POPF) was defined according to the 2016 definition proposed by the International Study Group of Pancreatic Fistula (ISGPF) [14]. Post-pancreatectomy haemorrhage was defined as intra-abdominal or intestinal bleeding according to the criteria of the International Study Group of Pancreatic Surgery (ISGPS) [15]. Delayed gastric emptying was defined according the criteria of the ISGPS [16]. Reoperation was defined as any surgical procedure performed in the first 30 postoperative days or before discharge from the hospital. Length of hospital stay (LOS) was calculated as the interval from the day of surgery to the date of discharge. The total cost of the surgical procedures was calculated in Euros and included pre-, intra-, and postoperative costs for the reference year 2019. The initial purchase expense of the laparoscopic system was excluded. Preoperative costs regarded the hospitalisation costs; intraoperative costs included operative theatre cost/hour and device costs; and postoperative costs included the hospitalisation costs, postoperative imaging studies, nutritional support, surgical reoperation or interventional postoperative procedures, intensive care unit admission expenses, and readmission costs.

### Statistical analysis

Statistical analyses were carried out using the Statistical Package for the Social Science (SPSS, Chicago, IL), version 13 on a personal computer. All the categorical variables were described as frequencies and percentages, while the continuous variables were reported as means with standard deviation. Comparison of the groups was carried out using the Fischer’s exact test, Student’s *t* test, and Pearson chi square test. Two-tailed *P* values less than 0.05 were considered statistically significant. The propensity score matching analysis used relevant variables with the aim of equating the complexity of the surgical cases. The relevant variables were patient-related (gender, age, comorbidities, ASA score, BMI, previous abdominal surgery) and tumour-related (tumour size, tumour site, and malignancy). Two propensity score matching comparisons were carried out: CLDP versus TLDP and CLDP versus ODP. A matched group of patients was created with a 1:2 ratio in both the PSM analyses. The PSM method is closest to the neighbourhood method having a caliper width of 0.20. Standardised mean difference (SMD) was used to assess the balance of the clinical backgrounds between the two groups. An SMD < 0.2 indicated very small differences between the means (this implied that optimal balance regarding a variable was generally achieved), an SMD between 0.2 and 0.8 indicated medium differences (this implied that fairly sufficient balance regarding a variable was generally achieved), and SMD > 0.8 indicated considerable differences (this implied that poor balance regarding a variable was generally achieved).

## Results

### Unmatched population

Two hundred and five patients underwent DP for body-tail pancreatic tumours from January 2005 to January 2021: 105 (51.2%) ODPs, 81 (39.5%) TLDPs, and 19 (9.3%) CLDPs. The reasons for conversion to an open procedure were the following: tumour close to major vessels (7 cases, 36.8%), oncological concerns (4 cases, 21.1%), bleeding (4 cases, 21.1%), adhesions (2 cases, 10.5%), and poor visualisation of the tumour (2 cases, 10.5%). It should be pointed out that the conversion was due to the proximity of the tumour to major vessels (< 1 cm), even if it was always detected preoperatively by abdominal CT scan. The comparison regarding the relevant variables of CLDP versus TLDP and CLDP versus ODP is summarised in Table 1. The patients undergoing CLDP had a significantly higher BMI and more frequent comorbidities with respect to TLDP (28.4–6.2- versus 25.6–4.2 kg/m^2^; P = 0.017 and 16, 84.2% versus 45, 55.6%; P = 0.035, respectively). Open distal pancreatectomy was preferred in patients who had undergone previous abdominal surgery and in those who had tumours located in the neck-body of the pancreas (71 (67.6%) versus 8 (42.1%); *P* = 0.041 and 89 (84.2%) versus 10 (52.6%); *P* = 0.003, respectively). The comparisons regarding operative findings, postoperative outcomes, and total costs are summarised in Table 2. The patients who underwent CLDP had a significantly longer operative time (314 ± 72 versus 235 ± 69 min; *P* < 0.001), more frequent postoperative morbidity (12 (63.2%) versus 40 (49.4%); *P* = 0.009), CR-POPF rates (11 (57.9%) versus 18 (22.2%); *P* = 0.004), and increased costs (19,760 ± 7504 versus 14,989 ± 4670 Euros; *P* = 0.014) with respect to TLDP. Converted laparoscopic distal pancreatectomy had a significantly longer operative time (314 ± 72 versus 260 ± 74 min; *P* = 0.006), less frequent postoperative morbidity (12 (63.8%) versus 94 (89.5%); *P* = 0.009), higher readmission rates (5 (26.3%) versus 8 (7.6%); *P* = 0.029), and increased costs (19,760 ± 7504 versus 19,092 ± 7256 Euros; *P* < 0.001) with respect to ODP.

**Table 1:** Unmatched population: comparison of relevant variables between CLDP versus TLDP and CLDP versus ODP

Relevant variables	All patients (*n*, % or mean, SD)			TLDP vs CLDP		ODP vs CLDP	
	ODP (*n* = 105)	TLDP (*n* = 81)	CLDP(*n* = 19)	*P* value	SMD	*P* value	SMD
Gender				0.304	0.321	0.804	0.110
M	50 (54.9)	31 (38.3)	10 (52.6)				
F	55 (52.4)	50 (61.7)	9 (47.4)				
Age (years)	64.4 (12.9)	59.9 (15)	62.5 (13)	0.429	0.178	0.554	0.147
BMI (kg/m^2^)	25.9 (5.0)	25.6 (4.2)	28.4(6.2)	**0.017**	0.626	0.114	0.481
ASA score				0.214	0.452	0.753	0.117
I	2 (1.9)	5 (6.2)	0 (0.0)				
II	31 (29.5)	41 (50.6)	7 (36.8)				
III	69 (65.1)	35 (43.2)	12 (63.2)				
IV	3 (2.9)	0 (0.0)	0 (0.0)				
Comorbidities				**0.035**	0.799	0.561	0.282
No	25 (23.8)	36 (44.4)	3 (15.8)				
Yes	80 (76.2)	45 (55.6)	16 (84.2)				
Previous abdominal surgery				0.310	0.326	**0.041**	0.581
No	34 (32.4)	35 (43.2)	11 (57.9)				
Yes	71 (67.6)	46 (56.8)	8 (42.1)				
Extended resection				0.458	0.149	0.314	0.182
No	77 (73.3)	73 (90.1)	16 (84.2)				
Yes	28 (26.7)	8 (9.9)	3 (15.8)				
Malignant tumours				**0.028**	0.451	0.512	0.118
No	69 (65.7)	66 (81.5)	11 (57.9)				
Yes	36 (34.3)	15 (18.5)	8 (42.1)				
Tumour size (mm)	39 (29.0)	32 (25.0)	29 (28.0)	0.668	0.111	0.175	0.338
Tumour site				0.293	0.324	**0.003**	0.888
Neck-body	89 (84.8)	54 (66.7)	10 (52.6)				
Tail	16 (15.2)	27 (33.3)	9 (47.4)				

Legend: *TLDP* total laparoscopic distal pancreatectomy, *ODP* open distal pancreatectomy, *CLDP* converted laparoscopic distal pancreatectomy, *SMD* standardised mean difference, *BMI* body mass index, *ASA* American Society of Anesthesiologists

Bold values indicate the results significantly different between the groups compared

**Table 2:** Unmatched population: operative findings, postoperative outcomes,and total costs of CLDP versus TLDP and CLDP versus ODP

Parameters	All patients (*n*, % or mean, SD)			TLDP vs CLDP		ODP vs CLDP	
	ODP (*n* = 105**)**	TLDP (*n* = 81)	CLDP (*n* = 19)	*P* value	SMD	*P* value	SMD
Operative time (min)	260(75)	235(69)	314 (72)	** < 0.001**	1.132	**0.006**	0.717
Blood loss (ml)	230 (305)	123 (94)	161(92)	0.121	0.40	0.057	0.467
90-day mortality				*		*	
No	105 (100)	81 (100)	19 (100)				
Yes	0 (0.0)	0 (0.0)	0 (0.0)				
Postoperative morbidity (C-D score)				**0.009**	0.439	**0.009**	0.299
No	11 (10.5)	41 (50.6)	7 (36.8)				
I	25 (23.8)	13 (16.1)	0 (0.0)				
II	56 (53.3)	18 (22.2)	10 (52.6)				
III	11 (10.5)	9 (11.1)	1 (5.3)				
IV	2 (1.9)	0 (0.0)	1 (5.3)				
V	0 (0.0)	0 (0.0)	0 (0.0)				
Severe postoperative morbidity (C-D > 2)				1.000	0.033	1.000	0.101
No	92 (87.6)	72 (88.9)	17 (89.5)				
Yes	13 (12.4)	9 (11.1)	2 (10.5)				
CR-POPF				**0.004**	0.866	0.068	0.557
No	70 (66.7)	63 (77.8)	8 (42.1)				
Yes	35 (33.4)	18 (22.2)	11 (57.9)				
POPF grade C				0.345	0.822	0.153	*
No	105 (100.0)	80 (98.8)	18 (94.7)				
Yes	0 (0.0)	1 (1.2)	1 (5.3)				
PPH				0.396	0.377	1.000	0.158
No	84 (80.0)	74 (91.4)	16 (84.2)				
Yes	21 (20.0)	7 (8.6)	3 (15.8)				
DGE				0.576	0.203	1.000	0.058
No	100 (95.2)	78 (96.3)	18 (94.7)				
Yes	5 (4.8)	23 (3.7)	1 (5.3)				
Reoperation				1.000	0.093	1.000	0.058
No	100 (95.2)	76 (93.8)	18 (94.7)				
Yes	5 (4.8)	5 (6.2)	1 (5.3)				
Readmission				0.306	0.397	**0.029**	0.808
No	97 (92.4)	69 (85.2)	14 (73.7)				
Yes	8 (7.6)	12 (14.8)	5 (26.3)				
Length of hospital stay (days)	16 (9)	10 (4)	13 (6)	0.096	0.432	0.322	0.024
Total cost (euro)	19,092 (7256)	14,989 (4670)	19,760 (7504)	**0.014**	0.899	** < 0.001**	0.091

Legend: *TLDP* total laparoscopic distal pancreatectomy, *ODP* open distal pancreatectomy, *CLDP* converted laparoscopic distal pancreatectomy, *SMD* standardised mean difference, *C-D* Clavien-Dindo, *CR-POPF* clinically relevant-postoperative pancreatic fistula, *PPH* post-pancreatectomy haemorrhage, *DGE* delayed gastric emptying. *Not computable

Bold values indicate the results significantly different between the groups compared

### Matched population

Using propensity score matching, three groups of patients were created with a 1:2 ratio, and well-balanced groups of 19 CLDPs, 38 TLDPs and 38 ODPs were compared. The samples compared were similar, and no differences were observed in the relevant variables. The SMD was always between 0.2 and 0.8, indicating a fairly sufficient balance of the groups (Table 3).

**Table 3:** Matched population: comparison of relevant variables between CLDP versus TLDP and CLDP versus ODP

Relevant variables	Propensity-matched patients (n, % or mean, SD)			TLDP vs CLDP		ODP vs CLDP	
	ODP (*n* = 38)	TLDP (*n* = 38)	CLDP (*n* = 19)	*P* value	SMD	*P* value	SMD
Gender				0.092	0.553	0.781	0.117
M	22 (57.9)	11 (28.9)	10 (52.6)				
F	16 (42.1)	27 (71.1)	9 (47.4)				
Age (years)	66.6 (11.2)	62.9 (13)	62.5 (12.6)	0.906	0.746	0.648	0.349
BMI (kg/m^2^)	25.8 (4.9)	26.5 (4.7)	28.4 (6.2)	0.258	0.344	0.115	0.491
ASA score				0.450	0.213	0.453	0.188
I	0 (0.0)	0 (0.0)	0 (0.0)				
II	13 (34.2)	18 (47.4)	7 (36.8)				
III	22 (57.9)	20 (52.6)	12 (63.2)				
IV	3 (7.9)	0 (0.0)	0 (0.0)				
Comorbidities				1.000	** < **0.001	0.735	0.194
No	8 (21.1)	6 (15.8)	3 (15.8)				
Yes	30 (79.9)	32 (84.2)	16 (84.2)				
Previous abdominal surgery				0.278	0.351	1.000	< 0.001
No	22 (57.9)	16 (42.1)	11 (57.9)				
Yes	16 (42.1)	422 (57.9)	8 (42.1)				
Extended resection				0.145	0.071	0.787	0.394
No	25 (65.8)	33 (86.8)	16 (84.2)				
Yes	13 (34.2)	5 (13.2)	3 (15.8)				
Malignant tumours				0.152	0.387	0.560	0.155
No	25 (65.8)	29 (76.3)	11 (57.9)				
Yes	13 (34.2)	9 (23.7)	8 (42.1)				
Tumour size (mm)	39 (26)	35 (28)	29 (28.0)	0.495	0.195	0.189	0.386
Tumour site				0.065	0.671	0.781	0.117
Neck-body	22 (57.9)	30 (78.9)	10 (52.6)				
Tail	16 (42.1)	8 (21.1)	9 (47.4)				

Legend: *TLDP* total laparoscopic distal pancreatectomy, *ODP* open distal pancreatectomy, *CLDP* converted laparoscopic distal pancreatectomy, *SMD* standardised mean difference, *BMI* body mass index, *ASA* American Society of Anesthesiologists

Operative findings, postoperative outcomes, and total cost after the propensity score matching analysis of CLDP versus TLDP and CLDP versus ODP are reported in Table 4. Propensity-matched patients who underwent CLDP had a significantly longer operative time (315 ± 72 versus 238 ± 68 min; *P* < 0.001), increased blood loss (161 ± 92 versus 108 ± 57 ml; *P* = 0.032), and total costs (19,760 ± 7506 versus 16,044 ± 5242 Euros; *P* = 0.034) with respect to TLDP. Propensity-matched patients undergoing CLDP had a significantly longer operative time (315 ± 72 versus 239 ± 61 min; *P* < 0.001), less frequent postoperative morbidity (12 (63.8%) versus 31 (81.6%); *P* = 0.050), and higher readmission rates (5 (26.3%) versus 2 (5.3%); *P* = 0.035) with respect to ODP. The reasons for readmission of the patients who underwent CLDP were the following: POPF grade B (treated with computed tomography (CT)-guided drainage) (3 cases); pleural effusion (medical treatment) (1 case), and nausea and vomiting (medical treatment) (1 case).

**Table 4:** Matched population: operative findings, postoperative outcome, and total cost of CLDP versus TLDP and CLDP versus ODP

Parameters	Propensity-matched patients (*n*, % or mean, SD)			TLDP vs CLDP		ODP vs CLDP	
	ODP (*n* = 38)	TLDP (*n* = 38)	CLDP (*n* = 19)	*P* value	SMD	*P* value	SMD
Operative time (min)	239(61)	238(68)	315 (72)	** < 0.001**	1.096	** < 0.001**	1.157
Blood loss (ml)	204 (256)	108 (57)	161(92)	**0.032**	0.753	0.351	0.198
90-day mortality				*	*	*	*
No	38 (100)	38 (100)	19 (100)				
Yes	0 (0.0)	0 (0.0)	0 (0.0)				
Postoperative morbidity (C-D score)				0.195	0.259	**0.050**	< 0.001
No	7 (18.4)	16 (42.1)	7 (36.8)				
I	13 (34.2)	5 (13.2)	0 (0.0)				
II	14 (36.8)	13 (34.2)	10 (52.6)				
III	**3 (7.9)**	**4 (10.5)**	**1 (5.3)**				
IV	1 (2.5)	0 (0.0)	1 (5.3)				
V	0 (0.0)	0 (0.0)	0 (0.0)				
Severe postoperative morbidity (C-D > 2)				1.000	** < **0.001	1.000	< 0.001
No	34 (89.5)	34 (89.5)	17 (89.5)				
Yes	4 (10.5)	4 (10.5)	2 (10.5)				
CR-POPF				0.099	0.563	0.086	0.602
No	26 (68.4)	25 (65.8)					
Yes	12 (31.6)	13 (34.2)	11 (57.9)				
POPF grade C				0.333	*	0.333	*
No	38 (100.0)	38 (100.0)	18 (94.7)				
Yes	0 (0.0)	0 (0.0)	1 (5.3)				
PPH				0.389	0.432	1.000	0.102
No	31 (81.6)	35 (92.1)	16 (84.2)				
Yes	7 (18.4)	3 (7.9)	3 (15.8)				
DGE				1.000	0.239	1.000	< 0.001
No	35 (92.1)	36 (94.7)	18 (94.7)				
Yes	3 (7.9)	2 (5.3)	1 (5.3)				
Reoperation				1.000	0.239	1.000	0.397
No	37 (97.4)	35 (92.1)	18 (94.7)				
Yes	1 (2.6)	3 (7.9)	1 (5.3)				
Readmission				0.478	0.355	**0.035**	1.026
No	36 (94.7)	32 (84.2)	14 (73.7)				
Yes	2 (5.3)	6 (15.8)	5 (26.3)				
Length of hospital stay (days)	14 (7)	12 (6)	13 (6)	0.491	0.215	0.457	0.202
Total cost (euro)	17,704 (5589)	16,044 (5242)	19,760 (7504)	**0.034**	0.612	0.299	0.327

Legend: *TLDP* total laparoscopic distal pancreatectomy, *ODP* open distal pancreatectomy, *CLDP* converted laparoscopic distal pancreatectomy, *SMD* standardised mean difference, *C-D* Clavien-Dindo, *CR-POPF* clinically relevant-postoperative pancreatic fistula, *PPH* post-pancreatectomy haemorrhage, *DGE* delayed gastric emptying. *Not computable

Bold values indicate the results significantly different between the groups compared

## Discussion

Recent studies have shown that converted cases had increased overall postoperative morbidity, surgical site infections, prolonged length of stay, and 30-day mortality as compared to completed TLDPs, whereas they could be associated with similar outcomes when compared with planned ODPs [3, 7–10]. The current study, which included patients who underwent both laparoscopic and open approaches, showed, by using a propensity score matching analysis, that a converted laparoscopic distal pancreatectomy was related to worse outcomes with respect to patients who completed a laparoscopic distal pancreatectomy and to similar results with respect to patients who underwent planned ODP. In particular, CLDP had significantly increased operative time, blood loss, and total costs with respect to TLDP. Regarding the comparison with ODP, operative time and readmission rate were significantly increased in CLDP, while the postoperative morbidity rate was significantly in favour of CLDP. It should be noted that the differences between CLDP and TLDP mainly regarded the operative findings and the total cost of the procedures, whereas the postoperative outcomes were similar. On the other hand, CLDP and ODP differed mainly in postoperative outcomes, while the total costs were similar. However, it should be noted that the severe postoperative complications were not significantly different. Thus, ODP had increased minor complications, mainly surgical infections with respect to CLDP. The readmission rate was significantly higher in CLDP with respect to ODP; however, readmission was usually related to a POPF which was, for the most part, due to the pancreatic texture. In summary, there were no clinically relevant differences between the groups compared.

The present study used a propensity score matching analysis to equate surgical complexity and to obtain well-balanced groups and, subsequently, reliable and robust results. Interestingly, before PSM analysis, some differences in relevant variables were present in the population analysed. In particular, the conversion rate to an open procedure was significantly higher in patients with a high BMI and an increased comorbidity rate, whereas ODP was preferred in patients having a tumour located in the body of the pancreas and in those patients who had undergone previous abdominal surgery.

By applying the PSM analysis, the data pointed out the importance of the effect of conversion and the need for some comments regarding the potential merits and risks of the laparoscopic approach. The impact of the converted cases seemed to suggest the importance of an adequate patient selection for the laparoscopic approach in order to prevent conversion and worse outcomes and avoid useless costs, even if, in the present study, the costs for ODP and CLDP were similar (Table 4). However, in selected cases, the open approach would be preferable. The choice of the proper approach, minimally invasive or open, is difficult; in the current literature [1], the open approach is planned in selected cases, but the laparoscopic technique seems preferable, even if the conversion rate is high. However, several studies have identified and assessed the preoperative predictors of conversion, such as patients with vascular proximity (< 1 cm) of the tumour on preoperative imaging, undergoing a subtotal pancreatectomy, tumour located in the body of the pancreas, preoperative findings of malignancy, resection extending to the neighbouring organs, and surgeon expertise, which would aid in the proper selection of an operative approach [3–7]. To emphasise this aspect, the ongoing DIPLOMA trial (ISRCTN44897265; www.e-mips.com) has stated that tumour involvement or the abutment of major vessels (celiac trunk, mesenteric artery and portomesenteric vein) were considered to be exclusion criteria for the laparoscopic approach. On the other hand, Lof et al. [3] have suggested that only emergency conversion, mainly due to bleeding, was related to worse outcomes. Thus, it can be assumed that the potential advantages of starting with a minimally invasive approach, even in the event of open conversion, have to be considered, namely, easier dissection and enhanced visualisation. In summary, the indications for LDP should be carefully assessed by an experienced laparoscopic surgeon capable of obtaining all the advantages of the minimally invasive approach (easier dissection and enhanced visualisation), performing, if necessary, an early conversion with the aim of avoiding an emergency conversion. However, it should be noted that the indication for an open approach should be considered in difficult cases.

The present study has some limitations, mainly, the retrospective design, partially mitigated by a prospectively maintained database, and the small sample size in a single tertiary centre. However, it should be pointed out that, to overcome the selection bias in retrospective studies, the most effective method (PSM analysis) of obtaining well-balanced groups to compare was carried out which allowed obtaining reliable results. In addition, the time interval considered in the present study was broad. Other biases may have occurred during this period, such as the different expertise of the surgeons, the difficulty of the surgery, and the different postoperative protocols which could have been utilised during the study period (referring to the total cost analysis). Finally, the results of the present study may also have been affected by the surgeons’ learning curves. However, the surgical team learning curve would mitigate this limitation.

## Conclusion

In conclusion, despite its limitations, by applying PSM analysis to obtain reliable results, the present study reported the impact of conversion with respect to both TLDP and ODP. Total laparoscopic distal pancreatectomy was superior regarding operative findings and total costs with respect to CLDP. On the contrary, ODP had a higher postoperative morbidity rate and a lower readmission rate with respect to CLDP. However, it should be pointed out that the reasons for the readmission of patients who underwent CLDP were mainly related to POPF grade B, which is usually due to pancreas texture. Thus, a careful assessment of the patients who should undergo a laparoscopic approach, the importance of identifying preoperative risk factors for conversion, and the possibility that an open procedure may be preferred seem to be mandatory. However, the results of the present study, regarding the impact of conversion with respect to both TLDP and ODP, suggest starting the majority of distal pancreatectomies using a minimally invasive approach, and performing an early conversion, if necessary.
